# Elevated resting heart rate predisposes metabolic syndrome in women rather than in men: a 15-year prospective study

**DOI:** 10.1186/s12872-015-0104-3

**Published:** 2015-09-30

**Authors:** Si Wang, Kai Liu, Xin Zhang, Qingtao Meng, Yong Wang, Shixi Wan, Xiaoping Chen

**Affiliations:** Department of Cardiovascular Medicine, West China Hospital, Sichuan University, Chengdu, 610041 China

**Keywords:** Resting heart rate, Metabolic syndrome, Sympathetic activity, Gender differences

## Abstract

**Background:**

Increasing evidences have indicated that there are gender differences in the prevalence of metabolic syndrome(MS), but the mechanism is uncertain.

**Methods:**

A total of 711 subjects aged 35–65 years accepted health examinations both in 1992 and 2007. Since 114 subjects had MS and 7 had heart disease at baseline, they were excluded from the analysis. Therefore, 590 subjects with complete data (male: 61.5 %) were available and analysed. After the relationship between gender and incident MS at follow-up was tested, these subjects were categorized into four groups according to the baseline resting heart rate(RHR) classified by genders. Trend tests of MS incidences across the four groups of resting heart rate were conducted by Cochran-Armitage tend tests in both men and women. Additionally, three logistic regression models were used to estimate the effects of RHR on the new onset of MS by taking RHR as a continuous variable(per 4 beats/min elevation).

**Results:**

Gender(women) itself was an independent risk factor for incident MS at follow-up(OR = 2.64, 1.33–5.23, *P* = 0.005). The incidences of MS according to the RHR categories showed a statistical linear trend in women(*P* for trend = 0.018) rather than in men(*P* for trend = 0.194). The ORs[95 % confidence intervals(CIs)] of MS for each 4 bpm elevation in RHR was 1.18(1.03–1.36)(*P* = 0.020) in a univariate model, 1.20 (1.04–1.38) (*P* = 0.011) adjusted for age and health related behaviors only and 1.23(1.06–1.43)(*P* = 0.007) adjusted for age, health related behaviors and pre-existing components of MS in the baseline in women. Otherwise, RHR did not have any significant associations with incident MS in men neither in a univariate model nor in multivariate models.

**Conclusions:**

In this study, elevated RHR is correlated with the development of MS in women rather than in men.

## Background

The metabolic syndrome(MS) is defined as a combination of interrelated cardiovascular disease(CVD) risk factors including abdominal obesity, hyperglycemia, raised blood pressure, elevated triglyceride and decreased high-density lipoprotein cholesterol [1]. Patients with the metabolic syndrome are at more than twice the risk of developing CVD over the next 5–10 years compared with those without the syndrome [1, 2]. Obviously, along with the modern life style changing, MS is becoming a major global public health problem which actually can be prevented. Accordingly, it is important to find the probably risk factors of the aggregation of multiple CVD risk factors so as to prevent the development of MS.

Increasing evidences have indicated that there are gender differences in the prevalence of MS [3–6]. The prevalence of MS in postmenopausal women is higher than that in men at the same age, but the mechanism is uncertain. Maybe there are some differences in the pathogenesis of the aggregation of multiple CVD risk factors in different genders. Several short-term follow-up studies [7, 8] have indicated that resting heart rate(RHR), which is a widely accepted indicator of sympathetic activity, is a significant risk factor of incident MS in men but not in women, but they could not give a good explanation for the differences. On the other hand, some studies [9–11] have found that body reactions of sympathetic activation are elevated significantly in women after menopause, which means, prolonging the follow-up period may get a different result of the relationship between RHR and incident MS in women. In order to clarify these issues, we utilized our data of a long-term longitudinal follow-up cohort in our Chinese people. We also used RHR as the indicator of sympathetic activity.

## Methods

### Participants and risk factors measurements

The study population was a part of the Chinese Multi-provincial Cohort Study(CMCS) in an urban community located in Chengdu, Sichuan province. A total of 711 subjects aged 35–65 years in the baseline accepted health examinations both in 1992(baseline) and 2007(follow-up) for cardiovascular disease (CVD) risk factors according to the World Health Organization-Multinational Monitoring of Trends and Determinants in Cardiovascular Diseases (WHO-MONICA) [12]. This includes a standardized questionnaire, physical examinations and laboratory tests. The questionnaire included demographic characteristics, smoking status, alcohol consumption, physical activity, and medical history of diabetes, hypertension and heart disease (coronary artery disease, heart failure or arrhythmia), but the details of anti-hypertensive medication were unavailable. Physical examinations included blood pressure, heart rate, height, weight and waist circumference measurements. After at least 5-min of rest in seated position, resting heart rate was obtained by pulse palpation over a 30-s period while seated, then blood pressure was recorded from the right arm as an average value of two consecutive blood pressure readings using a regular mercury sphygmomanometer in the same position. Laboratory tests included fasting plasma glucose (FPG), fasting serum total cholesterol (TC), low-density lipoprotein cholesterol (LDL-C), high-density lipoprotein cholesterol (HDL-C), and triglyceride (TG) by overnight blood samples drawn from the antecubital vein. FPG, TC and TG were all determined using the enzymatic method, while the concentration of LDL-C was estimated using the Friedewald formula and HDL-C was measured using the phosphotungstic acid/magnesium chloride precipitation method [13]. Since 114 subjects were identified MS and seven subjects with heart disease in 1992, they were excluded from the analysis. On the other hand, since diabetes mellitus(DM) was just a component of MS, we haven’t excluded it all at baseline. Therefore, 590 subjects with complete data (male: 61.5 %) were available and analysed. This study was approved by Ministry of Health of China, as well as by the Ethics Committee of West China Hospital of Sichuan University. All participants provided written informed consent.

### Related definitions

The diagnosis of the metabolic syndrome was based on the newly modified National Cholesterol Education Program (NCEP) ATP III Criteria as proposed by the updated American Heart Association (AHA)/National Heart, Lung, and Blood Institute (NHLBI) scientific statement [1]. Accordingly, any 3 of the 5 following items constituted diagnosis of metabolic syndrome: (1) waist circumference ≥80 cm in Asia women or ≥90 cm in Asia men; (2) fasting triglyceride concentration ≥1.7 mmol/L or on drug treatment for elevated triglycerides; (3) high density lipoprotein cholesterol (HDL-C) concentration <1.3 mmol/L in women or <1.0 mmol/L in men or on drug treatment for reduced HDL-C; (4) blood pressure ≥130 mmHg (systolic) or 85 mmHg (diastolic) or on antihypertensive drug treatment in a patient with a history of hypertension; and (5) fasting plasma glucose ≥5.6 mmol/L or on drug treatment for elevated glucose. Smoking was defined as average cigarette consumption ≥1/day. Alcohol intake was defined as average intake of alcohol ≥50 g/week. Physical activity was defined as exercise one or more times per week, at least 20 min for each time.

### Statistical analysis

Continuous data were presented as means ± standard deviations (SDs), and categorical variables were presented as frequencies and percentages.

Differences of baseline characteristics between participants classified by genders were tested by Student’s t tests for continuous data and chi-square tests for categorical data.

The difference in incidence of MS at follow-up between two genders was tested by chi-square test too and the relationship between gender and incident MS at follow-up was then tested by a multiple logistic regression analysis adjusting for age, RHR, health related behaviors(including smoking, drinking, and exercise) and pre-existing components of MS, by taking gender as a categorical variable(0 = men, 1 = women).

After that, subjects were categorized into four groups according to the baseline resting heart rate, that is, group 1(≤70 beats/min), group 2(71–80 beats/min), group 3(81–90 beats/min) and group 4(>90 beats/min) in different genders. Trend tests of MS incidences across the four categories of resting heart rate were conducted by Cochran-Armitage tend tests in both men and women. Additionally, to assess the effects of resting heart rate on the new onset of MS in different genders, three logistic regression models were used to estimate the odds ratios (ORs) and 95 % CI values, by taking RHR as a continuous variable(per 4beats/min elevation). In these analyses, model 1 was a univariate model, included only RHR as the independent variable; model 2 was adjusted for age and health related behaviors(included smoking, drinking, and exercise) and model 3 was adjusted for age, health related behaviors and pre-existing components of MS. Cochran-Armitage trend tests were conducted using SAS software, version 6.12 (SAS Institute Inc, Cary, NC) while the other statistical analyses were conducted using SPSS software version 18.0(SPSS, Chicago, IL). The statistical significance was defined as *P* <0.05.

## Results

### Baseline characteristics of the participants in 1992 classified by genders

Table 1 shows the baseline characteristics of all participants in 1992 classified by genders. Of the 590 enrolled participants, there were 227(38.5 %) women and 363 men (61.5 %). The baseline average age was 46 ± 6 years in women and 49 ± 6 years in men. The women participants were younger than men. The percentages of smoking and drinking in women were significantly lower than in men, and the percentages of obesity, raised blood pressure(BP), raised TG and decreased HDL in women were significantly higher than in men.

**Table 1:** Baseline characteristics of the participants in 1992 classified by genders

	Women	Men	*P* value
Number(%)	227.0(38.5)	363.0(61.5)	–
Age(years)	46 ± 6	49 ± 6	<0.001
Height	154.6 ± 5.4	165.2 ± 5.7	<0.001
Weight	54.9 ± 6.7	62.6 ± 8.0	<0.001
Waist(cm)	72.2 ± 6.3	77.4 ± 7.4	<0.001
BMI(kg/m^2^)	23.0 ± 2.4	22.9 ± 2.7	0.923
Smoking(n(%))	1(0.4)	228(62.8)	<0.001
Drinking(n(%))	6(2.6)	210(57.9)	<0.001
Exercise(n(%))	46(20.3)	78(21.5)	0.723
RHR(beats/min)	79.9 ± 8.3	80.2 ± 9.4	0.632
SBP(mmHg)	109.9 ± 12.9	113.4 ± 12.8	0.001
DBP(mmHg)	71.3 ± 8.3	73.2 ± 8.5	0.011
FPG(mmol/L)	4.31 ± 1.12	4.26 ± 0.74	0.479
TG(mmol/L)	1.86 ± 0.73	2.04 ± 0.86	0.011
TC(mmol/L)	4.50 ± 0.80	4.42 ± 0.70	0.200
LDL(mmol/L)	2.32 ± 0.81	2.22 ± 0.78	0.103
HDL(mmol/L)	1.30 ± 0.24	1.24 ± 0.22	<0.001
Obesity(n(%))	25(11.0)	16(4.4)	0.002
RBP(n(%))	21(9.3)	64(17.6)	0.005
RFPG(n(%))	7(3.1)	18(5.0)	0.271
RTG(n(%))	97(42.7)	202(55.6)	0.002
DHDL(n(%))	113(49.8)	57(15.7)	<0.001
MS components	1.16 ± 0.73	0.98 ± 0.76	0.001

Data are presented as means ± SD or number (percentage)

*BMI* Body mass index, *RHR* Resting heart rate, *SBP* Systolic blood pressure, *DBP* Diastolic blood pressure, *FPG* Fasting plasma glucose, *TG* Triglyceride, *TC* Total cholesterol, *LDL* Low-density lipoprotein, *HDL* High-density lipoprotein, *RBP* Raised blood pressure, *RFPG* Raised fasting plasma glucose, *RTG* Raised triglyceride, *DHDL* Decreased high-density lipoprotein

### Gender difference in incidence of MS at follow-up

The incidence of MS was 30.8 % in women and 17.6 % in men over the 15-year follow-up period. The difference in incidence of MS between two genders was statistically significant(*P* <0.001). Multiple logistic regression analysis showed that, after adjusting for age, RHR, health related behaviors(including smoking, drinking, and exercise) and pre-existing components of MS, gender(women) itself was also an independent risk factor for incident MS at follow-up(OR = 2.64, 1.33–5.23, *P* = 0.005).

### Incidences of MS according to the RHR categories at baseline classified by genders

**Fig. 1:**
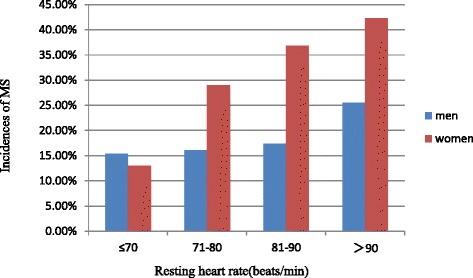
Incidences of MS according to the RHR categories at baseline. women P for trend = 0.018; men P for trend = 0.194

As shown in Fig. 1, the incidences of MS over the 15-year follow-up period in women according to the baseline RHR categories were 13.0, 28.9, 36.8 and 42.3 %, in 23, 121, 57 and 26 participants respectively, and there was statistical linear trend through the four groups (*P* for trend = 0.018) for the 15 year period. In addition, the incidences of MS over the 15-year follow-up period in men according to the baseline RHR categories were 15.4, 16.1, 17.4 and 25.5 %, in 52, 155, 109 and 47 participants respectively. There was a raising trend across the four groups for the 15 year period, but the linear trend didn’t reach statistical significant (*P* for trend = 0.194).

### Logistic regression analyses for the relationship of RHR at baseline and incident MS at follow-up in different models classified by genders

Table 2 shows the results of logistic regression analyses for the relationship of RHR and incident MS in different models. In women, each 4beats/min elevation in resting heart rate was associated with an approximately 1.2-fold increase in the risk of developing metabolic syndrome in a univariate model(model 1, OR = 1.18, 1.03–1.36, *P* = 0.020) and a multi-variate model adjusting for age and health related behaviors only(model 2, OR = 1.20, 1.04–1.38, *P* = 0.011). Additionally, after we adjusted for age, health related behaviors and pre-existing components of MS (model 3, OR = 1.23, 1.06–1.43, *P* = 0.007), the association between the risk of developing metabolic syndrome and tachycardia was still existed.

**Table 2:** Logistic regression analyses for the relationship between RHR at baseline and incident MS at follow-up in different models classified by genders

	Women			Men		
	OR	95 % CI	*P* value	OR	95 % CI	*P* value
Model 1	1.18	1.03–1.36	0.020	1.08	0.97–1.21	0.177
Model 2	1.20	1.04–1.38	0.011	1.08	0.96–1.21	0.199
Model 3	1.23	1.06–1.43	0.007	1.08	0.96–1.22	0.218

Model 1: univariate model

Model 2: adjusted for age and health related behaviors(included smoking, drinking, and exercise)

Model 3: adjusted for age, health related behaviors and pre-existing components of MS

Otherwise, resting heart rate did not have any significant associations with incident MS in men neither in a univariate model nor in multivariate models.

## Discussion

In this 15-year prospective follow-up study, we found that elevated resting heart rate in the baseline is correlated to the development of metabolic syndrome in the follow-up in women rather than in men independent of age, health related behaviors and any of MS components. The incidence of MS increased with RHR elevated in women while not in men. Our findings were not entirely consistent with previous study results.

Some cross-sectional studies have already found a correlation between RHR and MS in different populations [14–18]. But they haven’t found gender differences in the relationship between RHR and MS. Just in consistent with previous studies [3–6], our study revealed that there is significantly gender difference in the prevalence of MS at follow-up, and the prevalence of MS in women was higher than that in men at 15-year follow-up. Maybe there are some differences in the pathogenesis of the aggregation of multiple CVD risk factors in different genders over time, so we think it is valuable to analysis separately in men and women in longitudinal follow-up studies. Just along with our opinions, Inoue et al. and Oda et al. confirmed gender differences in their longitudinal researches [7, 8]. Inoue et al. demonstrated that men with elevated baseline heart rates were more likely to experience metabolic syndrome than were those with normal heart rates while it was not true for female patients over the 5-year period follow up [7], and Oda et al. indicated that an increase in resting heart rate was a significant risk factor for MS in non-obese Japanese men but not in women through 3 years [8], but both of them could not give a good explanation for the differences. There was another prospective study that provided a different point of view. In this study, Zhang found that a high resting heart rate is independently associated with an increased risk of type 2 diabetes (an important component of MS) in women during a mean follow-up of 4.9 years [19]. Although this study was not a direct evidence to certify the relationship between RHR and MS in women, it at least demonstrated that gender differences in the relationship between RHR and MS may remain controversial.

Our study is the first long time follow-up study of the relationship between RHR at baseline and incident MS 15 years later. The relationship between RHR and incident MS was found in women, but not observed in men, and the association was still exist in women when age, health related behaviors and pre-existing components of MS were adjusted for. The results of the study are different in regard to gender differences in the relationship between RHR and incident MS compared with previous studies. It may be a chance result, but the participants in our study are not the same as previous studies and there are some rational explanations. First, the development of MS and its associated components are chronic processes and cannot come up in a short time. As a result, the relationship between baseline RHR and incident MS should be elucidated in a long observation time and maybe confused by some potential factors in a relatively short period of follow-up. Second, as influenced by different genetic backgrounds, life styles and social environment factors, there may be differences in the pathogenesis of MS in populations from different places. Third, we adopted waist measurement as the component of MS according to the newly published guideline [1], while some researches just used BMI [7], which may not be an appropriate indicator for MS. It is now widely accepted that insulin resistance is the most important component of MS. Elevated sympathetic activity could antagonise insulin’s effect on glucose uptake via effects on blood flow(sympathetically mediated vasoconstriction) in skeletal muscle, result in hyperinsulinemia and insulin resistance [20, 21]. But gender differences exist in the body reactions of sympathetic activation. Kneale et al. demonstrated that young women exhibit blunted vasoconstrictor responses to alpha-adrenergic stimulation compared to men [22], which may be related to the vasodilator effect of estrogen [23–28]. However, Vongpatanasin indicated that menopause can lead to enhanced alpha-adrenergic peripheral vasoconstriction both at rest and during exercise [9] and Sherwood et al. demonstrated that women exhibit altered sympathetic nervous system activity and a sustained increase in hemodynamic load after menopause [11]. Since the majority of the women participants entered in our studies at the beginning were in the perimenopause and in the elderly age at the follow-up, we consider that the 15-year follow-up may make the effect of sympathetic activation on metabolism more apparent in women. On the other hand, the relationship between RHR and incident MS was weaker in men than in women. Maybe it needed more men participants to get statistically significant, particularly when there was an increasing trend for men.

Our study has several limitations. First, we haven’t adopted electrocardiogram as the measurement of resting heart rate as most studies have. However, according to Zhang [19], pulse palpation over a 30-s is an accepted method too. Second, we haven’t completely eliminated the effect of drugs on resting heart rate, but we have excluded patients with heart disease in our study, those mostly needed drugs with impacts on resting heart rate. Third, we just used resting heart rate as the alternative indicator of sympathetic activity, which is easily obtained, like most studies, but it may not be an ideal surrogate for sympathetic activity. Forth, MS is just a complex multifactorial health problem, and it has limited practical utility as a diagnostic or management tool, but it is worthwhile to further elucidate the underlying pathways of the clustering of such a lot of risk factors.

## Conclusions

In conclusion, the present study reveals that elevated resting heart rate in middle age is an important risk factor for developing MS in old age in women but not in men. The inconsistency may be due to gender differences that exist regarding bodily reactions to sympathetic activation, but the mechanisms need to be further studied. On the other hand, it is encouraging to explore the potential of direct modulation of activity of the sympathetic nervous system as a therapeutic strategy to prevent the development of metabolic syndrome.
